# Endogenous emotion generation ability is associated with the capacity to form multimodal internal representations

**DOI:** 10.1038/s41598-018-20380-7

**Published:** 2018-01-31

**Authors:** Haakon Engen, Philipp Kanske, Tania Singer

**Affiliations:** 1 Department of Social Neuroscience, Max-Planck-Institute for Human Cognitive and Brain Sciences, Leipzig, Germany; 2 Medical Research Council Cognition and Brain Sciences Unit, University of Cambridge, Cambridge, UK; 3 Institute of Clinical Psychology and Psychotherapy, Department of Psychology, Technische Universität Dresden, Dresden, Germany

## Abstract

Training the capacity to self-generate emotions can be a potent “vaccine” against negative stressors and be an effective intervention for affective psychopathology. However, due to a lack of knowledge about sources of individual differences in generation abilities, it is unclear how to optimally design such interventions. We investigated one potential source of variation, namely preference for using different information modalities (*Visual Imagery, Auditory Imagery, Bodily Interoception*, and *Semantic Analysis)*. A representative sample of 293 participants self-induced positive and negative emotional states, freely choosing to use these modalities singly or in combination. No evidence was found for modality usage being associated with differential efficacy at generating of positive or negative emotion. Rather, usage of all modalities (except *Auditory Imagery)* predicted success at generation of both positive and negative emotional states. Increasing age predicted capacity to generate, especially negative, emotions. While no specific combinations of modalities were superior, the overall degree to which participants adopted multimodal implementations did predict generation efficacy. These findings inform interventions aimed at improving emotional self-generation, suggesting these must be mindful of individual differences in generation abilities and implementation tendencies, and that they should focus on enhancing the capacity to use multiple modalities.

## Introduction

Emotions are paradigmatically thought of as reactions to events in the external world. However, they frequently occur due to internal events, such as our own streams of thought or recalled memories. A unique aspect of such emotional states is that they can be volitionally generated, e.g. by controlled recall of memory for emotionally charged events. Such endogenous generation of emotion (EGE) elicits subjective, neural and psychophysiological responses, that strongly resemble emotional responses to exogenous stimuli^1–5^, and it has been suggested that the generation of such states plays an important role in coping and emotion regulation^6–10^. Supporting this conjecture, evidence is accumulating that disturbances of EGE might be diagnostic of affective psychopathology^11–13^. Interestingly, recent research suggests that the capacity to endogenously generate positive emotional states can be trained, and that such training might serve as a “vaccine” against the impact of negative life events^7,8^. Thus, devising optimal interventions aimed at enhancing emotion generation abilities shows promise as a psychological intervention that can both increase resilience in non-clinical populations and directly counteract affective psychopathologies such as depression^7,14^.

In order to optimise such intervention, it is important to have a clear picture of how emotion generation abilities vary and what individual factors might predict this variation. Current research suggests that the modality of information processing employed in EGE is one such factor. For instance, studies show that visual mental imagery of emotionally charged events is particularly effective at eliciting both positive and negative emotional states^5,9,11,13,15^, possibly due to mental imagery resulting in perception-like experiences, that in turn drive emotion generation mechanisms in a similar way to actual perception^11,13^. Accordingly, studies show stronger experienced emotion following processing of emotional scenarios using imagery in comparison to semantic processing, which does not have a clear perceptual component^5,7,16,17^. Thus, this line of evidence suggests that intervention should take the form of training to use visual mental imagery^7,14^.

However, this conclusion must be tempered by several limitations to previous research. For one, studies that have investigated the efficacy of modalities have, to our knowledge, exclusively compared single modalities (e.g. visual mental imagery vs. semantic processing^7^). However, considering that emotional experiences are inherently multimodal, including clear perceptual, semantic, and interoceptive aspects^18,19^, there is reason to expect that endogenous emotional representations are similarly multimodal^2,20^. This suggest that combinations of modalities might be more efficacious than any single modality in isolation. Thus, it is possible that information modalities might interact in determining the emotional impact of a representation. Similarly, it is plausible that there is significant individual variation in the capacity to draw on information in different modalities, which is an important datum for the future development of tailored EGE training interventions.

**Figure 1:**
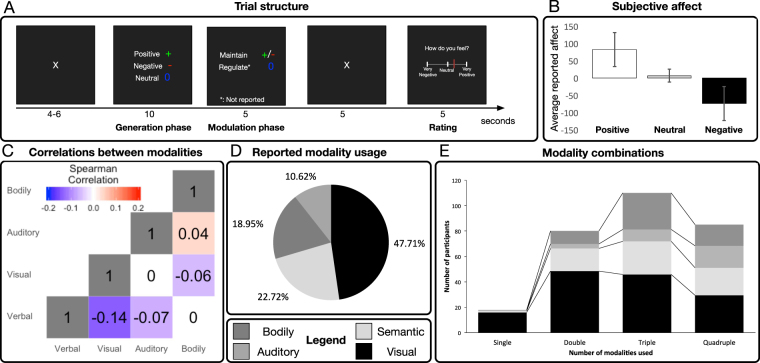
(**A**) Schematic representation of the experiment. Following an automatized procedure where participants trained and selected how to generate emotional states, participants were asked to generate positive, negative and neutral states. Each trial started with a fixation cross (4–6 s.). Participants were then presented with cues indicating what emotional state to generate (green plus-sign = generate positive, red minus-sign = generate negative, blue zero = generate neutral) for 10 seconds. For the emotional conditions, the cue then either changed to a blue zero indicating that participants should down-regulate their generated states or remained the same as in the generation phase indicating that they should maintain the emotional state for another 5 seconds. Only this Maintain-condition was the topic of the current study. This was followed by a 5 second fixation cue, and a 5 second bipolar subjective affect rating. (**B**) Effect of generation instructions on subjectively reported affect. (**C**) Spearman correlations between modalities. (**D**) Modality usage reported by participants to generate emotions. (**E**) Reported combinations of modalities as a function of number of modalities participants reported using.

The current paper sought (1) to explore individual differences in preference for using different information processing modalities in EGE and (2) whether these differences were predictive of the capacity to generate emotion. To do this we performed a reanalysis of behavioural data from a recent neuroimaging study^2^ using a recently developed paradigm designed to provide a maximally naturalistic assay of EGE. Participants (*N* = 293) self-generated positive and negative emotional states in a completely cue-based paradigm (see Fig. 1A), using one or more of four information processing modalities (*Semantic Analysis, Visual Imagery, Auditory Imagery*, and *Bodily Interoception*) in whichever combination and degree they believed would facilitate successful emotion generation. These modalities cover most information sources known to partake in emotional experiences^18,19^, while simultaneously representing discrete modes of processing that are amenable to both instruction and combination. In a previous investigation of the current dataset we have shown^2^ that this task elicits strong changes in subjective, psychophysiological and neural markers of emotion. Moreover, in the previous investigation we showed that self-reported modality usage correlated with activation of functional neural networks known to be associated with primary processing of these modalities. In the current investigation we sought to expand on these findings and explore whether usage of different modalities was associated with differential efficacy in the generation of emotional states. Importantly, we address a shortcoming of most previous studies in having participants generate both positive and negative emotion, allowing us to differentiate general from valence-specific effects.

The current research had two distinct objectives. First, we aimed to address the complete lack of descriptive research on what information processing modalities individuals spontaneously use when they engage in EGE. To do this, we investigated overall usage of different modalities and their combinations, so as to provide a descriptive account of what modalities and combinations of modalities participants preferred in generating emotional states. Our second objective was to establish whether specific modalities or combinations of modalities were associated with different levels of EGE efficacy. Thus, we investigated how reported modality usage was associated with EGE efficacy, defined as the strength of reported subjective affect when participants generated positive and negative emotion relative to a neutral baseline. As discussed above, a major omission of previous work has been its focus on comparing the efficacy of single information processing modalities in isolation. Therefore, we were particularly interested in identifying interactions between modalities. We investigated this by allowing participants to freely combine modalities as they saw fit, and tested whether we could find evidence of modalities interacting in determining generation success. Similarly, as the majority of previous work has focused on single valences when examining efficacy of modalities, we wanted to see if any observed modality effects were valence-specific or not. This is important as most current interventions emphasise improved generation of specifically positive affect as the “active ingredient” in EGE based trainings^7,21^. To this end, several regression models were specified separately investigating the effect of reported modality usage on general (i.e. both positive and negative emotion) and relative (i.e. positive relative to negative emotion) generation efficacy.

Due to the lack of previous research investigating the topic, we were unable to formulate concrete *a priori* hypotheses regarding distribution of preferences. For efficacy, based on previous research^7,17,22^ we expected to find that *Visual Imagery* should show increased efficacy relative to *Semantic Analysis*, and that *Visual Imagery* should overall be the most efficacious modality. Moreover, we expected that *Visual Imagery* should be more effective than *Semantic Analysis* at generating positive, relative to negative, emotional states.

## Results

### Subjective ratings

The main outcome measures in the current analyses were the average of subjective emotion reports given after participants generated and maintained positive and negative emotional states over the course of 15 seconds (Maintain conditions; see Fig. 1A) or actively tried to maintain a neutral emotional state. Ratings were analyzed using paired *t* tests of the average reported affect in each condition (see Fig. 1B). Relative to the Neutral condition (mean = 7.64, SD = 19.16), participants reported increased affect for positive (mean = 83.23, SD = 48.87; paired *t* (292) = 27.24, *p* < 0.001, CI (95%) = 70.13/81.05) and negative (mean = −73.13, SD = 49.11; paired *t* (292) = −27.94, *p* < 0.001, CI (95%) = −86.46/−75.08) Maintain conditions. In addition to subjective report, we also recorded galvanic skin response of acceptable quality as measure of emotional arousal in a subset of 225 participants. In a previous paper examining the current dataset^2^ we reported subjective ratings of experienced emotion predicted skin conductance levels. We investigated if this relationship was modulated by modality usage using linear mixed modelling and found no effects. See *Supplementary Analyses* for more detail.

### Operationalizing emotion generation efficacy

Generation efficacy scores were created by subtracting average ratings in the Neutral baseline condition from averages in the Positive and Negative Maintain conditions. Spearman rank correlation revealed a strong association between the ability to generate positive and negative emotions (*r*_*s*_ = 0.69, *p* < 0.001; This correlation was also observed for the direct comparison of positive and negative generation scores (r_s_ = 0.66, p < 0.001); see Supplemental Figure S1). To account for shared variance and differentiate average generation efficacy from relative capacity to generate positive and negative emotion, we defined two efficacy scores: Average generation efficacy was operationalised as the average of positive and negative generation efficacy. Conversely, relative generation efficacy was operationalised as the difference score between positive and negative efficacy. These scores were not significantly correlated (*r*_*s*_ = 0.05, *p* = 0.37), suggesting they correspond to different aspects of emotion generation abilities.

### Characterizing the preference structure of emotion generation modalities

We next sought to establish how our participants implemented emotion generation. This was assessed based on participants’ self-report of what modalities they had used during the experiment, as assessed by a post-experiment questionnaire with 9-point Likert scales anchored with “Not used at all” and “Used a great deal” and labeled with the modality name (see *Methods* for more detail). Additionally, participants were given the option to report whether they had used “Other” modalities (that had not been previously trained; see *Methods* for description of training procedures) and to describe them in writing. 14 participants reported using “Other” modalities, but on closer inspection, all except one of the participants’ descriptions closely matched our primary modalities and were therefore included in the primary scores by averaging (see Supplementary Table S1). The final participant reported using several of the primary modalities to a large degree and so their “Other” score was ignored. To control for individual differences in rating tendency we calculated the proportion of reported usage of each modality relative to the sum-total of usage reports. (See *Supplementary Methods* for a compendium of calculations and Supplementary Figure S1 for illustration of raw rating reports). Pairwise Spearman correlations between modalities are reported in Supplementary Table S2 and Fig. 1C, and reveal that modalities are largely uncorrelated, with the exception of a significant anti-correlation between *Semantic Analysis* and *Visual Imagery*. However, this correlation did not survive Bonferroni correction (adjusted *p* = 0.08). Reported modality usage is shown in Fig. 1D. *Visual Imagery* was the most used (47.71%), followed by *Semantic Analysis* (22.72%), *Bodily Interoception* (18.95%) and *Auditory Imagery* (10.62%).

Next, we investigated how participants combined modalities. Usage of a given strategy was operationalised as a response other than “*Did not use at all*”. Results (Supplementary Table S3 and Fig. 1E) showed that most participants used two or more modalities, with the largest proportion combining three modalities, primarily by combining other modalities with *Visual Imagery*. Thus, these results demonstrate that participants largely preferred multi-modal implementations of emotion generation, but that *Visual Imagery* tended to serve as “core” for these implementations.

### Efficacy of emotion generation modalities

**Figure 2:**
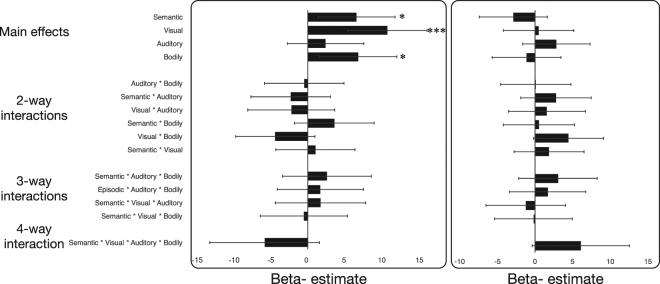
Plots of beta-estimates from multiple regression models investigating the effect of modality usage on emotion generation efficacy. (**A**) Relationship between modality usage and average emotion generation efficacy. (**B**) Relationship between modality usage and the relative ability to generate positive and negative emotion. Error bars = 95% confidence intervals. *p < 0.05, **p < 0.01, ***p < 0.001.

The efficacy of different modalities was investigated in two separate multiple regression models for average (Positive *and* Negative) and relative (Positive *minus* Negative) generation efficacy. Aside the predicted variable, models were identical. Modality usage scores were Z-scored and entered as continuous, interacting predictors. Age and gender was included as control variables. Results from these analyses are reported in Fig. 2 and Supplementary Table S4. Average generation efficacy was found to be significantly predicted by usage of all modalities except *Auditory Imagery*. Additionally, we observed an effect of age, such that older individuals were more effective. When analyses were repeated for relative efficacy, no significant effects were observed except, again, an effect of age, which was associated with increased efficacy at generating negative, relative to positive, emotions.

To investigate the relative strengths of the effects of modality of average generation efficacy, we used the *multcomp* R package to perform pairwise tests for significant difference between parameters estimates of effect for each modality (see Supplementary Table S5), revealing no significant differences between modalities.

Summarising, usage of *Visual imagery, Semantic Analysis* and *Bodily Interoception* (but not *Auditory Imagery*) was associated with higher generation efficacy. No evidence was found for differential efficacy for positive and negative emotion generation. Moreover, results suggest that these modalities were similarly efficacious when considered alone, and that no combination of these modalities was particularly associated with increased efficacy.

### Degree of multimodal implementation as a predictor of generation efficacy

Despite this lack of differential efficacy of modalities alone or in combination, most participants reported adopting multiple techniques. This could suggest that multimodal generation implementation is a predictor of generation efficacy in and of itself. The preceding analyses would not detect this, as significant interactions would only be in evidence if particular combinations of modalities would show differential efficacy. We therefore departed from our analysis plan and investigated this directly by entering the sum of reported usage scores across modalities as a predictor, and age and gender as nuisance covariates in a multiple regression model. This revealed that generation efficacy in general (*b* = 2.16, t = 3.95, p < 0.001) significantly predicted overall reported usage of modalities. No effect was found for relative efficacy (*b* = −0.20, *t* = −42, *p* = 0.67). These results suggest that degree of multimodal implementation was associated with increased generation efficacy in a similar way for positive and negative emotion.

## Discussion

The current research had two main objectives: First, we sought to provide a descriptive account of the information modalities individuals use to implement endogenous generation of emotion (EGE) when given free choice. Second, we wanted to explore whether usage of different modalities was associated with efficacy at generating emotional states. In a newly developed, minimally constrained paradigm, 293 participants generated positive and negative emotion, freely choosing to use one or more of four information modalities known to be associated with emotional experiences (*Visual Imagery, Semantic Analysis, Auditory Imagery, Bodily Interoception*), as well as self-defined modalities. This allowed us to map preference for the four modalities in the normal population, while at the same time exploring prevalence of other modalities. Further, by comparing reported modality usage with success at generation, we could determine how usage of different modalities affected generation efficacy.

Overall, we found that participants were successful at generating both positive and negative emotional states, as indicated by subjective report. Moreover, we found that participants used all four of the modalities to achieve these states, and that they tended to use them in combination. Minimal usage of self-formulated modalities was reported. This suggests that our results adequately sample the space of naturally occurring EGE modalities, allowing us to achieve our first objective of providing a descriptive account of what combination of modalities participants prefer using to implement EGE. *Visual Imagery* was the most frequently used modality, followed by *Semantic Analysis*, *Bodily Interoception*, and *Auditory Imagery*. Most participants reported using a combination of modalities to generate emotion, most frequently using other modalities in adjunct to *Visual Imagery* (see Supplementary Table S2 and Fig. 1D), with *Semantic Analysis* and *Bodily Interoception* being used in approximately equal measure.

Our second objective was to investigate if particular modalities or combinations of modalities were associated with higher EGE efficacy. Based on previous research^7,17,22^, we expected *Visual Imagery* to be superior to *Semantic Analysis*, with no clear hypotheses for the other modalities. Contrary to this, we did not find that any specific modality or combination of modalities outperformed others: *Semantic Analysis*, *Visual Imagery* and *Bodily Interoception* usage had comparable positive effects on generation efficacy (see Supplementary Table S5), and there was no evidence for interaction effects suggesting particular combinations of modalities were associated with differential efficacy for either average or relative generation (see Fig. 2). Despite this, most participants spontaneously adopted multimodal implementations (see Fig. 1E), and in a follow-up analysis we found that the degree to which they did so directly predicted generation success. Moreover, contrary to previous studies^7,8^, our findings suggest that modalities have similar efficacy for generation of both positive and negative emotional states. This goes against our predictions, as well as previous findings showing valence-differentiation for mental imagery and semantic analysis.

While our efficacy findings suggest equivalence of modalities, this must be tempered by the observation that participants seldom elected to use *Semantic Analysis* or *Bodily Interoception* alone, and that *Visual Imagery* usage far outstripped the other modalities. This suggests that emotion generation efforts, these were effectively rooted in *Visual Imagery*. One possible explanation for this can be found in the constructive memory literature, where it has been shown that the generation of internal simulations consists of at least two distinct phases^23^, consisting of an initial recall of key information features of simulated events that “seed” subsequent elaboration of this information by spreading activation of related information. Possibly, a similar operation sequence pertains to EGE, with visual imagery serving as the “seed” information to which semantic, and bodily information is added. Importantly, this account differs from the perceptual explanations for modality efficacy proposed in previous literature^11,13^. These accounts propose that the key determinant for the degree to which an internal representation causes emotional reactions is its resemblance to perceptual experiences. Our findings suggest it is not the perceptual fidelity of the representation that is important, but rather the richness of information marshalled. From this perspective, the determining factor for the emotional potency of an internal representation is how closely the internal representations approximate – or simulate–the multi-modal, first-person experience of emotional experiences^18,24^, irrespective of the precise modality employed or the degree to which these modalities resemble actual perception. It should be noted that such a simulation account effectively is an extension of the perceptual account, shifting the focus from sensory information to all aspects of the emotional experience. Consistent with this, it should be noted that the studies showing superiority of mental imagery relied on inductions that require the construction of episodic simulations of hypothetical events^5,7,16,17^. Importantly, such imagery is inherently multimodal, involving the integration of a wide range of scenario-relevant information, including perceptual, semantic, spatial, and even bodily aspects of events^25^. Thus, the reported superiority of mental imagery to semantic analysis might be explained as stemming from comparing the effect of a multimodal with a mono-modal implementation. This has potential important implications for interventions aimed at improving the capacity to generate emotional states^7,8^, suggesting that such training should aim at training the capacity to use multiple information modalities rather than training usage of any specific modality. However, support for this conclusion is limited by methodological constraints in the current study, as modality usage was only measured using retrospective self-report. The aim of this relatively unconstrained sampling approach was to give a measure of how endogenous emotion generation is spontaneously effected in the normal population. Future research should directly test this accounts by taking a more constrained approach in which participants are systematically assigned to different modalities or combinations of modalities.

### Limitations and future directions

The current results found no evidence for *Auditory Imagery* being an efficacious modality for emotion generation, as it was both seldom used, and (uniquely) showed no correlation with generation success. However, the relative inefficacy and lack of use of the *Auditory Imagery* modality could be due to the loud scanner environment interfering with the implementation of the modality. Moreover, from qualitative debriefings during piloting and during the main experiment, it appeared that musical expertise was an important factor in determining whether participants elected to employ *Auditory Imagery*.

This observation points to two important topics for future research, namely the impact of context and individual differences in determining the efficacy and the susceptibility of different information processing modalities in facilitating different forms of endogenous emotion generation. This would also be a step in further specifying the factors that might influence the efficacy and learnability of potential interventions aimed at increasing emotion generation skills, moving towards tailoring such interventions to the individual. An important aspect the current results do not address is the impact of content on the efficacy of different modalities. For instance, while we did not find evidence for differential efficacy of modalities during the generation of positive versus negative emotion, it is possible that different contents of information processing mask such effects, such that negative, past-focused semantic analysis (i.e. rumination) might be more effective than future-focused imagery of possible negative events. Identifying the dimensions of information processing determining the efficacy of different modalities in facilitating endogenous generation of emotion is an important topic for future work. Similarly, an interesting question for future research is to investigate how the superiority of heteromodal generation is related to individual differences in emotional processing in general. Future work should investigate whether the capacity to endogenously generate emotions might be associated with how vividly and nuanced individuals experience emotions. This can further the enhance our understanding of how modality-specific information processing interacts with core affective processes to create emotional experiences^26^.

Another important topic the current study cannot address is how different modalities might have sequential carry-on effects. Thus, for instance, it is possible that using a given modality (for instance *Semantic Analysis*) can cause the occurrence of emotionally charged *Visual Imagery*, which in turn might be the actual cause of the emotional response. Future research could investigate this by including more detailed assessment of both usage of a given modality and their phenomenological consequences. A related limitation is that the employment of modalities was only assessed at the end of the experiment, in a manner that relies heavily on both participants’ introspective abilities to accurately assess and remember the degree to which they used each of the different modalities during the experiment. The time-constraints imposed by the data being acquired in the context of an MRI scanning session did not allow trial-wise assessment of generation implementation in the current experiment. Future research should include this, as this would provide better estimates of the efficacy of single modalities, as well as make possible the assessment of inter-individual differences in the stability of their ability and tendency to implement emotion generation.

An interesting observation in the current results is the effect of age on generation efficacy (Supplementary Table S4), such that increased age appears to be associated with increased ability to self-generate especially negative emotional states. This appears to be at odds with the growing literature that old age is associated with a so-called positivity effect, such that older individuals appears to have a stronger cognitive bias towards positive stimuli, especially in unconstrained experiments^27^. One possibility is that this discrepancy reflects a decreased tolerance for negative emotional experiences with age, resulting in greater subjective distress. Another possibility is that age is associated with greater willingness to confront negative emotional states. While these explanations are necessarily speculative, future research could clarify this by e.g. investigating whether this age effect is associated with laboratory measures of cognitive bias. Importantly, however, these findings provide important information for future EGE-based interventions, suggesting that age is could be a critical factor prediciting the ease with which EGE can be trained and how likely such training is to have an effect.

Finally, it should be noted that, while our findings support focusing on multimodality in potential clinical interventions, heed must be taken to the role different forms of information processing in different psychopathological conditions. For instance, it is commonly observed that mental imagery is neglected in the thinking processes of patients with depression, being replaced by thoughts with a verbal focus^11,28^. Similarly, anxiety-related pathologies are often characterised by an over-emphasis on bodily markers of emotion^29^. This suggests that that our findings on the relative efficacy of different modalities might not hold for psychopathological populations, and that there is likely to exist considerable individual differences in how effective these modalities are for emotion generation. Ultimately, this could suggest a diagnostic function of investigating the efficacy of different modalities for emotion generation. Future research should investigate this in more detail, as it is possible that a more complete understanding of fundamental disturbances of the processes and mechanisms of endogenous emotion generation in affective pathology would improve our understanding of the etiology of disorders, and, potentially, how such biases can be counteracted by targeted intervention.

## Methods

### Data availability

All data and *R* scripts to perform the reported analyses are available at OSF (https://osf.io/9zj5b/).

### Participants

Data was acquired in the context of an fMRI study, the neural data of which are reported in^2^. Participants were recruited in the context of the large-scale longitudinal ReSource Project^30^, with baseline data being used for the present study. Eligibility was determined using a screening procedure including SCID-I and II interviews performed by trained clinical psychologists, ensuring no ongoing mental health issues, and no life-time occurrence of psychotic or bipolar disorders, substance dependence, or any Axis-II disorders. For comparability, we employed the same sample in the current study as the previous fMRI study. Out of a recruited sample of 332, 305 participants completed the current paradigm. 5 participants were excluded due to missing data caused by e.g. technical difficulties. 4 participants reported difficulties during the experiment (e.g. nausea or sleepiness), and a further 3 participants were removed due to aberrant behavior suggestive of task non-compliance, such as having low or no variance in behavioral ratings. This left a final sample of 293 (170 female, mean age = 40.4, range: 20–55, SD = 9.3). The study was approved by the Research Ethics Committees of the University of Leipzig (number 376/12-ff) and the Humboldt University in Berlin (numbers 2013-02, 2013-29, and 2014-10) and was carried out in compliance with the Declaration of Helsinki. All participants gave written informed consent and were debriefed and paid after the study was completed.

### Training procedure

Before the experiment, participants underwent a supervised automated training session with two distinct stages. First, participants underwent a multimodal affect induction procedure that provided examples of high and low arousal positive and negative emotion. These inductions ensured that participants had homogenous representations of the target emotional states. Second, participants were introduced to four different means of emotion generation using one of four information processing modalities (*Visual Imagery, Semantic Analysis, Auditory Imagery*, and *Bodily Interoception*). In addition to corresponding to the induction procedure, these modalities were elected as they have been shown in previous literature to be effective means of self-inducing emotion^20,22,31,32^. Participants freely chose a modality or combination of modalities to generate emotions, and were also given the option to use self-formulated generation methods, and were requested to specify whether they would attempt to generate high- or low-arousal exemplars of positive and negative emotional states. Participants then trained generating positive and negative emotional states and were instructed to use the modality or combination of modalities that they experienced to best allow them to generate emotions in the main experiment. Further details of the training procedure are reported in the *Supplementary Methods*.

### Experimental procedure

A trial (Fig. 1A) started with a 4–6 second white fixation cross followed by a 10 second Generation phase, in which subjects were shown a cue indicating which emotional state to generate (Red minus = Negative, Green plus = Positive, Blue zero = Neutral). In the emotional conditions, a 5 second Modulation phase followed in which the instruction symbol remained the same (Maintain condition) or changed to a blue 0 indicating that subjects should down-regulate the emotional state they had generated (Regulate condition). The current study focused on the Maintain conditions to ensure that estimates of the efficacy of different modalities to generate emotions was not “polluted” by the influence of later regulatory efforts to change these generated emotions. In the Neutral condition, participants were instructed to actively maintain a neutral state of mind. There were 10 trials for each condition (50 in total) and their order was pseudo-randomised with no more than two consecutive repetitions of each condition.

After a 5 second fixation cross, a 5 second Visual Analogue Scale (VAS) ranging from “Extremely negative” via “Neutral” to “Extremely positive” (range:+/−250 from the neutral point) was presented. Initial cursor position was jittered randomly (range:+/−100 points relative to the Neutral point). Responses were given using the right index and middle finger. Participants were instructed to report their affective state as it was during report. Stimuli were back-projected in the MRI scanner using a mirror setup. Eyesight was corrected where appropriate. After the scanning session, participants filled out a questionnaire asking them to report which of the four modalities they had used to generate emotions during the experiment using 9-point Likert scales anchored with “Not used at all” and “Used a great deal” and labeled with the modality name (Verbal, Visual, Auditory, Bodily). The questionnaire also included an item for reporting usage of “Other” modalities, i.e. self-formulated techniques. If they reported using “Other” techniques, they were prompted to writing a short description what they did. These descriptions are reported in Supplemental Table S1.

## Electronic supplementary material

Supplementary materials
