# Supplementary material for: Endogenous emotion generation ability is associated with the capacity to form multimodal internal representations
Source: Sci Rep. 2018 Jan 31;8:1953. doi: 10.1038/s41598-018-20380-7 (PMC5792544; doi:10.1038/s41598-018-20380-7)
Supplement: Supplementary file 1 — Supplementary materials [file 41598_2018_20380_MOESM1_ESM.pdf]

Supplementary materials:

Endogenous emotion generation ability is associated with the capacity to form multimodal internal representations

Haakon Engen, Philipp Kanske & Tania Singer

Supplemental methods

1: Training procedure

Before scanning, participants underwent a supervised, automated training procedure. To ensure that participants had a homogenous representation of the affective states they were requested to generate, they first underwent a multimodal affect induction procedure combining emotional music, affectively charged pictures and verbal descriptions of bodily and psychological sensations associated with the different emotion states, ensuring that participants were not biased towards any particular information modality. Inductions aimed at inducing positive and negative emotional states of both high (e.g. happiness and fear) and low (e.g tenderness and sadness) arousal as well as give subjects an example of a neutral state by presenting them with emotionally neutral visual stimuli (neutral induction). Participants were then presented with a list of four emotion generation modalities with short descriptions, in addition to the option to self-specify a generation technique. Descriptions are presented below, translated from the original German:

Verbal[1]: Internal monologue. E.g. telling yourself things that you believe will make you feel different things. Instead of statements you can also tell yourself short stories. The important thing is that you use verbal abilities.

Visual[2]: Using visual imagery to imagine real or hypothetical situations or events that are emotional. Importantly, this technique is associated with a visual experience and not just e.g. retelling a story like in the Verbal).

Auditory[3]: As Visual, but with a focus on sounds, such as voices, music or other emotionally evocative sounds. Again, the important thing is that emotions should be elicited through auditory means.

Bodily[4]: Creating and/or amplifying bodily manifestations of emotions, such as breathing, facial expressions, muscular tension, heart rate, thereby changing ones emotional experience.

---

[1] Corresponds to the *Semantic Analysis* modality

[2] Corresponds to the *Visual Imagery* modality

[3] Corresponds to the *Auditory Imagery* modality

[4] Corresponds to the *Bodily Interoception* modality

Other: If you will be using a technique (either alone, or in combination with the others) that is not similar to any of the above, please indicate this by selecting E. You will be asked to describe this in writing.

The order of descriptions was counterbalanced. Participants were also given the option of using techniques or modalities that were not specified and asked to describe this in writing. Participants then chose which arousal level to generate (High/Low) separately for positive and negative emotion, and asked to select one or more techniques to employ. Three Generation trials identical to the experimental procedure followed in which participants generated positive, negative and neutral emotional states. After this, they were given the option to revise their initial selection of strategies in case they discovered the unsuitability of employed strategy and/or arousal level during training.

2: Calculation of modality usage scores

Preference structure of emotion generation was investigated by establishing usage controlled for overall reporting tendency by defining usage of a given modality as a proportion of overall reported modality usage as follows:
Let the four 9-point Likert ratings of modality usage collected post-experiment be **R1… R4**

Overall rating usage = ∑ (R1 … R4)
Proportionate modality usage = R1 … R4 / Overall rating usage

Example calculation:
Ratings recorded = (4, 2, 3, 0)
Overall rating usage = 9
Effective modality usage = (4/9, 2/9,3/9,0/9) = (44.4%, 22.22%, 33.33%, 0) = 100%

These effective modality scores were then averaged across participants, resulting in the proportion scores reported in Figure 1 C and D, and Table S3. See scripts available at https://osf.io/9zj5b/ for implementation.

3: Supplemental psychophysiological analyses

In Engen, Kanske, & Singer (2017) we described a trial-level relationship between skin conductance levels (SCL; a frequently used measure of emotional arousal) and ratings on the using mixed models. Ahe current work focuses on the relationship between average (trait-level) generation success and average reported usage of different modalities, we did not expect to find a relationship between modality usage and average SCR, especially since we did not find a relationship between averaged SCR and ratings in our previous paper. However, for completeness we have explored the relationship between modality usage and trial-level SCR responses using mixed modelling as implemented in the *lmer* R package. As in our previous study the tested model included fixed effects regressors for condition (Positive/Negative/Neutral) and Ratings (centred). Additionally, we included reported modality usage (centred), as well as nuisance covariates (age, gender). To test the effect of modality usage, the model also included subject-level random intercepts and random slopes for modality usage (note, results are very similar without these random slopes). All interactions were allowed, except between modality usage and ratings, both to avoid overfitting and because the results reported in the main text already show a relationship between modality usage and ratings. Thus, if reported modality usage specifically is associated with increased SCR responses during emotion generation one would therefore expect to see a [Modality usage] * Condition interaction. Results from this analysis are reported below, and revealed little evidence for a role of modality usage on subject SCL responses. There is a trend towards an interaction of Visual and Auditory and Visual and Semantic usage on SCL, but this is irrespective of experimental condition. Thus, overall, these results show that modality usage does not have a clear effect on SCL, and that any effect is mediated by increased subjective experience of emotion as indicated by ratings.

Variable	B	SE	Numer. df	Denom. df	F-value	p-value	Sig. Level
Effects of modality usage							
Semantic	0.000	0.00	1.00	144.82	0.01	0.93	
Visual	0.027	0.03	1.00	64.95	2.38	0.13	
Auditory	0.029	0.03	1.00	58.00	2.55	0.12	
Bodily	0.006	0.01	1.00	113.45	0.55	0.46	
Semantic:Visual	0.033	0.03	1.00	53.31	2.91	0.09	.
Semantic:Auditory	0.005	0.01	1.00	53.10	0.48	0.49	
Visual:Auditory	0.033	0.03	1.00	18.61	2.91	0.10	.
Semantic:Bodily	0.013	0.01	1.00	98.13	1.10	0.30	
Visual:Bodily	0.004	0.00	1.00	40.27	0.36	0.55	
Auditory:Bodily	0.001	0.00	1.00	42.77	0.12	0.73	
Semantic:Visual:Auditory	0.000	0.00	1.00	16.28	0.00	0.97	
Semantic:Visual:Bodily	0.001	0.00	1.00	36.61	0.08	0.78	
Semantic:Auditory:Bodily	0.008	0.01	1.00	40.56	0.69	0.41	
Visual:Auditory:Bodily	0.000	0.00	1.00	14.67	0.00	0.96	
Semantic:Visual:Auditory:Bodily	0.013	0.01	1.00	21.03	1.13	0.30	
Interactions of modality usage with condition							
Condition:Semantic	0.002	0.00	2.00	10645.47	0.09	0.92	
Condition:Visual	0.003	0.00	2.00	10645.37	0.14	0.87	
Condition:Auditory	0.008	0.00	2.00	10645.38	0.34	0.71	
Condition:Bodily	0.012	0.01	2.00	10645.34	0.50	0.61	
Condition:Semantic:Visual	0.002	0.00	2.00	10645.36	0.09	0.92	
Condition:Semantic:Auditory	0.001	0.00	2.00	10645.46	0.02	0.98	
Condition:Visual:Auditory	0.001	0.00	2.00	10645.26	0.05	0.95	
Condition:Semantic:Bodily	0.002	0.00	2.00	10645.31	0.07	0.93	
Condition:Visual:Bodily	0.004	0.00	2.00	10645.22	0.16	0.85	
Condition:Auditory:Bodily	0.005	0.00	2.00	10645.31	0.23	0.80	
Condition:Semantic:Visual:Auditory	0.002	0.00	2.00	10645.38	0.09	0.92	
Condition:Semantic:Visual:Bodily	0.003	0.00	2.00	10645.17	0.14	0.87	
Condition:Semantic:Auditory:Bodily	0.006	0.00	2.00	10645.36	0.26	0.77	
Condition:Visual:Auditory:Bodily	0.001	0.00	2.00	10645.17	0.06	0.94	
Condition:Semantic:Visual:Auditory:Bodily	0.001	0.00	2.00	10645.19	0.06	0.94	
Condition and ratings effects							
Condition	0.040	0.02	2.00	10646.74	1.73	0.18	
Ratings	0.045	0.04	1.00	10649.87	3.91	0.05	*
Condition:Ratings	0.211	0.11	2.00	10650.44	9.18	0.00	***
Control variables							
Age	0.004	0.00	1.00	174.97	0.38	0.54	
Gender	0.127	0.13	1.00	181.53	11.07	0.00	***

Relationship between modality usage and generation condition: Trial level analysis of effect of modality usage on SCL levels. . = p < .1, * = p < . 05, ** = p <. 01, *** = p <.001.

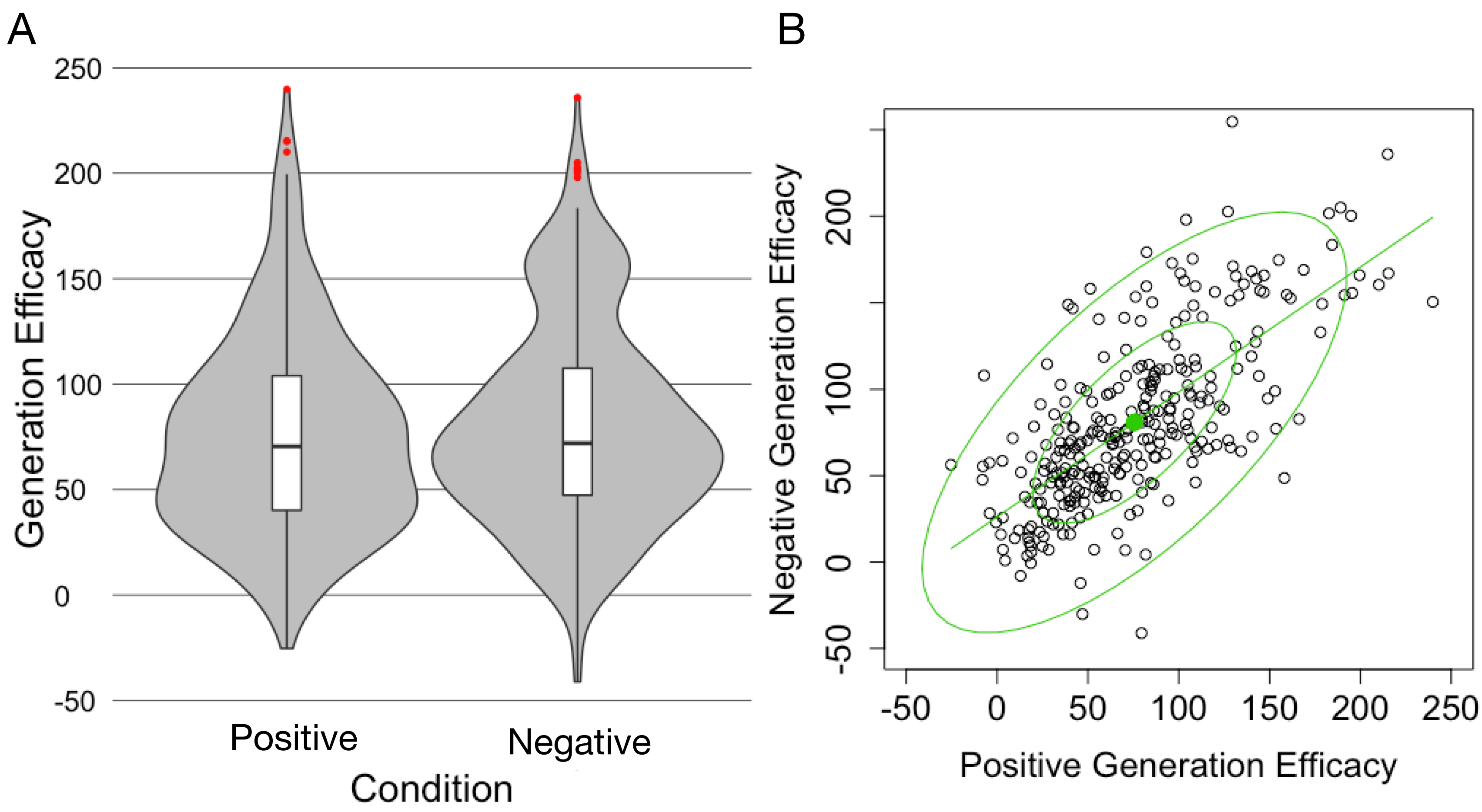

Figure S1: Distribution and correlation of generation efficacy scores. A) Violin and box plots of Positive and Negative Generation scores. Shaded area indicates distribution of data-points. Whiskers are largest value no further than 1.5 interquartile range. Red dots indicate data points in excess of this value. B) Scatterplot of the relationship between Positive and Negative Generation efficacy scores. Ellipses and line indicate 95% data-concentration and linear regression slope.

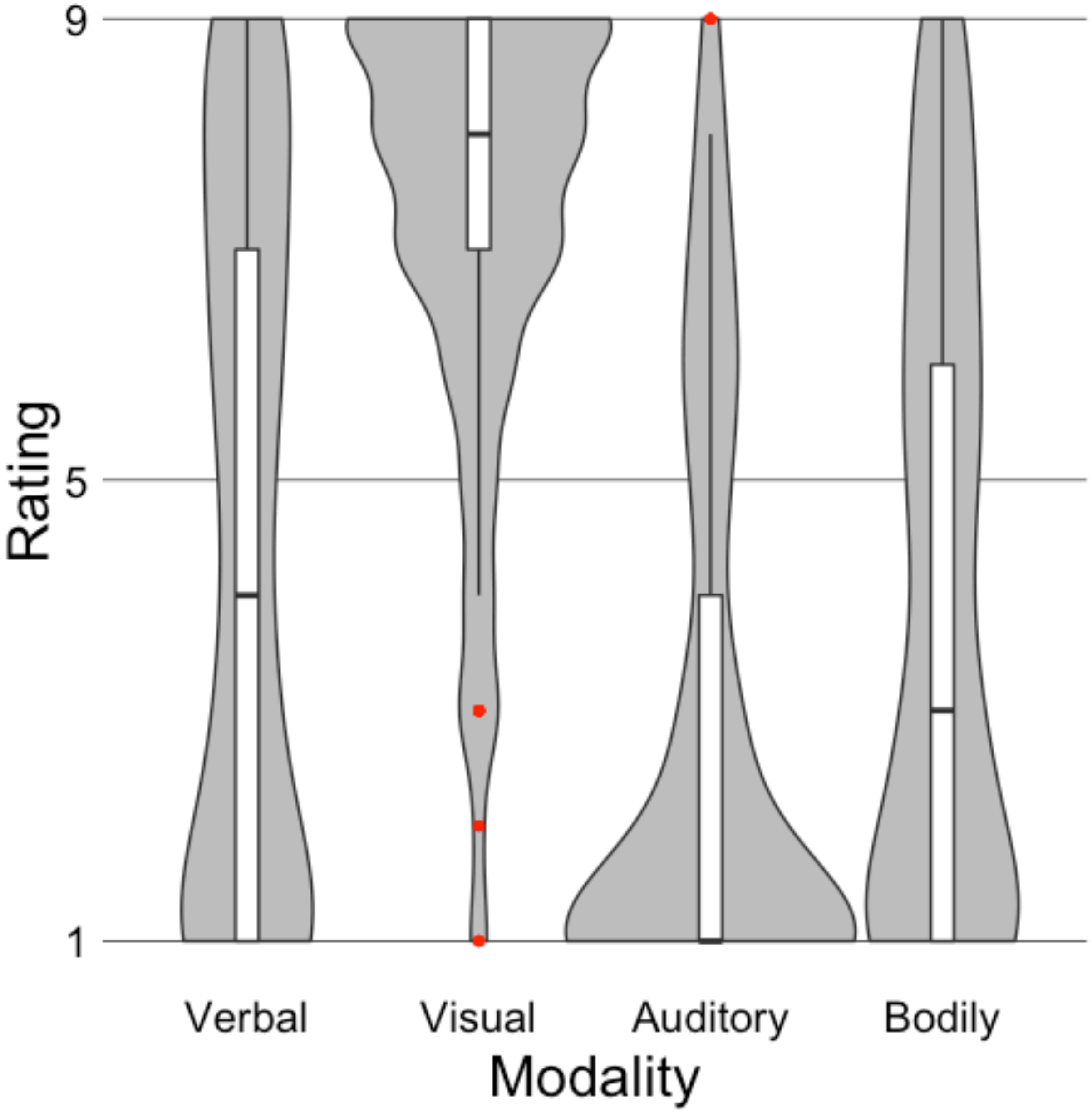

Figure S2: Violin and box plots of reported modality usage. Shaded area indicates distribution of data-points. Whiskers are largest value no further than 1.5 interquartile range. Red dots indicate data points in excess of this value.

Description	Assigned
Recalling past or future situations	Episodic Imagery
Optically imagining situations	Episodic Imagery
Memories	Episodic Imagery
Flying dream- Imagination is a mix of visual and bodily, and mostly mixed together	Episodic Imagery/ Bodily Interoception
Tactile memories	Bodily Interoception
Breathing	Bodily Interoception
Good and bad memories	Episodic Imagery
Memories of good and bad feelings	Episodic Imagery
Rekindling memories	Episodic Imagery
Going into the feeling	Not assignable
Concentrated on generating the feeling (feeling happy, positive, excited).<br>Tried to amplify feelings, memories and images that were evoked.	Episodic Imagery
Recalling memories, mentally playing guitar	Episodic Imagery/ Auditory Imagery
I thought of stories or events in my own past.	Episodic Imagery
Memories of feelings	Episodic Imagery

Table S1: Translated descriptions for each of the 14 participants reporting using "Other" modalities is reported, together with their assignment

	Semantic	Visual	Auditory	Bodily
**Semantic**	1.00			
**Visual**	-.14*	1.00		
**Auditory**	-0.05	-0.03	1.00	
**Bodily**	0.03	-0.07	0.07	1.00

Table S2: Correlation between modalities. Correlation coefficients = Spearman rho, * = $p < .05$, uncorrected

# of modalities	Combination	N	%	Proportional composition (%)			
				Visual	Semantic	Bodily	Auditory
Single	Visual	16	88,89	100	--	--	--
	Semantic	1	5,56	--	100	--	--
	Bodily	0	0,00	--	--	N/A	--
	Auditory	1	5,56	--	--	--	100
	**Total N**	18					
Dual	Visual/Semantic	39	48,75	59.48	40.52	--	--
	Visual/Bodily	28	35,00	66.54	--	33.46	--
	Visual/Auditory	10	12,50	65.91	--	--	34.09
	Semantic/Bodily	3	3,75	--	69.48	30.52	--
	Semantic/Auditory	0	0,00	--	N/A	--	N/A
	Bodily/Auditory	0	0,00	--	--	N/A	N/A
	**Total N**	79					
Triple	Visual/Semantic/Bodily	68	61,82	38.42	32.12	29.46	--
	Visual/Semantic/Auditory	15	13,64	48.49	26.01	--	25.50
	Visual/Bodily/Auditory	26	23,64	48.09	--	21.32	30.60
	Semantic/Bodily/Auditory	1	0,91	--	18.00	9.00	73.00
	**Total N**	108					
Quadruple	Visual/Semantic/Bodily/Auditory	83	100,00	34.68	25.61	19.43	20.28
	**Total N**	83					

Table S3: Usage and proportional composition of modality combinations as a function of number of modalities reported used. Number of participants reporting using each combination of modalities reported in the *N* column, whereas the self-reported degree to which each modality was used on average is reported in the *Proportional Composition* columns.

Variable	Model 1: Mean Generation Efficacy					Model 2: Positive - Negative Generation Efficacy				
	B	SE	t	CI (95%) -	CI (95%) +	B	SE	t	CI 95% -	CI 95% +
Variables of interest										
Semantic	6.61	2.66	2.48*	1.36	11.86	-2.88	2.30	-1.25	-7.42	1.65
Visual	10.80	2.75	3.92***	5.39	16.21	0.47	2.38	0.20	-4.21	5.14
Auditory	2.41	2.64	0.91	-2.79	7.61	2.82	2.28	1.24	-1.67	7.32
Bodily	6.83	2.68	2.54*	1.55	12.12	-1.13	2.32	-0.49	-5.70	3.44
Auditory * Bodily	-0.51	2.75	-0.19	-5.92	4.90	0.07	2.38	0.03	-4.61	4.75
Semantic * Auditory	-2.32	2.75	-0.84	-7.74	3.10	2.77	2.38	1.16	-1.91	7.45
Visual * Auditory	-2.26	3.01	-0.75	-8.19	3.67	1.56	2.60	0.60	-3.57	6.68
Semantic * Bodily	3.59	2.77	1.30	-1.85	9.03	0.50	2.39	0.21	-4.21	5.21
Visual * Bodily	-4.45	2.73	-1.63	-9.82	0.93	4.44	2.36	1.88	-0.21	9.09
Semantic * Visual	1.02	2.74	0.37	-4.37	6.41	1.84	2.37	0.78	-2.82	6.50
Semantic * Auditory * Bodily	2.59	3.07	0.84	-3.46	8.64	3.04	2.66	1.14	-2.20	8.27
Visual * Auditory * Bodily	1.68	2.98	0.56	-4.19	7.55	1.66	2.58	0.65	-3.41	6.74
Semantic * Visual * Auditory	1.73	3.12	0.56	-4.40	7.87	-1.24	2.69	-0.46	-6.54	4.07
Semantic * Visual * Bodily	-0.55	3.03	-0.18	-6.51	5.41	-0.22	2.62	-0.08	-5.38	4.94
Semantic * Visual * Auditory * Bodily	-5.87	3.79	-1.55	-13.33	1.59	6.07	3.28	1.85	-0.38	12.52
Control variables										
Age	7.91	2.66	2.97**	2.66	13.15	-6.76	2.30	-2.94**	-11.29	-2.23
Gender	-1.39	2.66	-0.52	-6.62	3.85	-4.30	2.30	-1.87	-8.83	0.23
Intercept	77.32	2.61	29.60	72.18	82.46	-4.09	2.26	-1.81	-8.54	0.35
Model R^2	0.064					0.035				

Table S4: Results from two multiple regression models investigating how modality usage predicts average (model 1; average of ratings in the positive and negative conditions, subtracting neutral condition) and relative (positive minus negative ratings) generation efficacy. * = p < .05, ** = p < .01, *** p < .001

Pairwise comparison of modality efficacies					
**Comparison**	B	SE	t	CI (95%) +	CI (95%) -
Semantic - Visual	-4.19	3.59	-1.17	-13.46	5.09
Semantic - Bodily	-0.22	3.76	-0.06	-9.93	9.48
Semantic - Auditory	4.20	3.54	1.19	-4.94	13.34
Visual - Bodily	3.96	3.62	1.10	-5.39	13.32
Visual - Auditory	8.39	3.79	2.21	-1.41	18.19
Bodily - Auditory	4.42	3.84	1.15	-5.50	14.35

Table S5: Pairwise comparison of parameter estimates of effect of modalities on general generation ability.
